# Consideration for the appropriate use of antimicrobials in long-term care wards

**DOI:** 10.1016/j.infpip.2026.100539

**Published:** 2026-04-12

**Authors:** M. Funato, R. Iwata, K. Yasuda

**Affiliations:** a Department of Pediatric Neurology, NHO Nagara Medical Center, Gifu, Japan; b Department of Pharmacy, NHO Nagara Medical Center, Gifu, Japan; c Department of Pediatric Surgery, NHO Nagara Medical Center, Gifu, Japan

**Keywords:** Antimicrobial stewardship, Long-term care wards, Multi-drug-resistant organism, Neuromuscular disorders, Severe motor and intellectual disabilities

## Abstract

**Background:**

Few studies have been conducted on the appropriate use of antimicrobials in long-term care wards, where patients with advanced neuromuscular disorders or severe motor and intellectual disabilities have been hospitalized for an extended period. This study aimed to confirm the clinical conditions requiring antibiotics in long-term care wards.

**Methods:**

Electronic medical records of patients who were administered intravenous infusions between 1^st^ January 2018 and 31^st^ December 2020 were retrospectively reviewed. The treatment methods were compared between two periods: from 1^st^ January 2018 to 30^th^ June 2019 (usual treatment group) and from 1^st^ July 2019 to 31^st^ December 2020 (optimal treatment group, based on the principles of antimicrobial stewardship).

**Results:**

Data from 65 patients (23 females, 42 males) with a mean age of 38 years and a median length of hospitalization stay of 14.5 years were analysed. A total of 206 intravenous infusions were administered, 86 in the usual treatment group and 120 in the optimal treatment group, of which 32 (37.2%) and 28 (23.3%) involved antibiotic therapy, respectively (*P* = 0.04). A significant difference was observed in the diagnosis of aspiration or respiratory tract infections (*P* < 0.01). Moreover, the peak level of C-reactive protein (CRP) during the event was identified as an important indicator.

**Conclusion:**

A comprehensive evaluation of patient status and CRP levels may help reduce inappropriate antibiotic use in long-term care wards.

## Introduction

The prevalence of multi-drug-resistant organism (MDRO) infections is a global problem that poses a significant risk to human health [1]. Therefore, appropriate prescription and optimal use of antimicrobials must also be addressed in not only acute-care settings but also non-acute-care settings [2]. MDRO infections in long-term care facilities for geriatric patients have been reported to be associated with more severe infections, increased risk and length of hospitalization, increased risk of death, and increased healthcare costs, and literature studies on antimicrobial stewardship in long-term care facilities are increasing [2,3]. However, the optimization of antimicrobial prescriptions in long-term care wards for patients with advanced neuromuscular disorders or severe motor and intellectual disabilities has yet to be reported. Herein, we describe the factors to consider for appropriate prescription and optimal use of antimicrobials in these long-term care wards.

## Methods

### Study design

Data were retrospectively collected from electronic medical records of patients who received intravenous infusion (including all events for one patient) and compared between two groups. From 1^st^ January 2018 to 30^th^ June 2019, patients were treated with standardized medical therapy (usual treatment group). From 1^st^ July 2019 to 31^st^ December 2020, patients were treated according to the principles of antimicrobial stewardship (optimal treatment group).

Specifically, based on the Japanese Ministry of Health, Labour and Welfare's guidelines for the appropriate use of antimicrobials [4], the following measures were taken to minimize antibiotic use: i) We first raised awareness of antimicrobial stewardship among our medical staff; ii) because some bacterial infections resolve spontaneously without the use of antibiotics, the focus of the infection was carefully determined; iii) when symptoms appeared and intravenous infusions were initiated, bacterial culture tests were performed, whenever possible; iv) regardless of whether antibiotics were used, vital signs were carefully monitored after the initiation of intravenous infusion; v) medical staff reviewed the appropriate use of antibiotics during daily conferences.

### Statistical analyses

Statistical comparison of the two groups involved the Fisher’s exact test for categorical variables shown in Table II and the unpaired *t*-test for quantitative variables shown in Table III, Table IV. Statistical significance was set at *P* < 0.05. All statistical analyses were performed using EZR (Easy R) (Saitama Medical Center, Jichi Medical University, Saitama, Japan) [5].

## Results

In this study, we analysed data from 65 patients between 1^st^ January 2018 and 31^st^ December 2020. The study included 23 women (35.4%) and 42 men (64.6%), with a median age of 38 (range, 8–62) years at the commencement of the study, and the mean age was 38.2 years. Six patients died; three from cardiac failure, one from renal failure, one due to kidney cancer, and one from multiple organ failure. In addition, two patients were transferred to another ward in Nagara Medical Center, and seven patients were newly admitted for long-term care or treatment. The proportion of patients with each underlying disease and condition is summarized in Table I.

**Table I:** Baseline clinical characteristics of patients included in the study

Variable	Level	Number of patients (%)
Sex	Female	23 (35.4)
	Male	42 (64.6)
Underlying disease	Duchenne muscular dystrophy	24 (36.9)
	Myotonic dystrophy type 1	5 (7.7)
	Fukuyama congenital muscular dystrophy	5 (7.7)
	Distal myopathy with rimmed vacuoles	1 (1.5)
	Spinocerebellar degeneration	2 (3.1)
	Angelman syndrome	1 (1.5)
	Rett syndrome	1 (1.5)
	Cerebral palsy caused by hypoxic–ischaemic encephalopathy, asphyxia, kernicterus, or unknown	20 (30.8)
	
	
	Sequelae of brain infarction, infection, diastematomyelia, or Reye's syndrome	6 (9.2)
Medical care		
Respiratory status	Spontaneous respiration	31 (47.7)
	Tracheal positive-pressure ventilation	21 (32.3)
	Non-invasive positive-pressure ventilation	11 (16.9)
	Tracheostomy without mechanical ventilation	2 (3.1)
Feeding method	Oral intake	30 (46.2)
	Tube feeding	35 (53.8)
Activities of daily living		
Eating	Dependent	63 (96.9)
	Needs help	2 (3)
	Independent	0 (0)
Toileting	Dependent	63 (96.9)
	Needs help	2 (3)
	Independent	0 (0)
Dressing	Dependent	65 (100)
	Needs help	0 (0)
	Independent	0 (0)
Bathing	Dependent	65 (100)
	Needs help	0 (0)
	Independent	0 (0)
Transferring	Dependent	62 (95.4)
	Needs help	3 (4.6)
	Independent	0 (0)
Hospitalization period (years)		
	>40	10 (15.4)
	30–39	8 (12.3)
	20–29	6 (9.2)
	10–19	16 (24.6)
	<9	18 (27.7)
	New	7 (10.8)

During the 3-year study period, we administered 206 intravenous infusions, which included 86 (41.7%) and 120 (58.3%) infusions in the usual and optimal treatment groups, respectively. Among the 206 intravenous infusions, 32 of 86 (37.2%) were treated with antibiotics in the usual treatment group and 28 of 120 (23.3%) in the optimal treatment group. In addition to a decrease in the number of antibiotic treatments, the frequency of antibiotic treatment was significantly different between the usual and optimal treatment groups (*P* = 0.04) (Table II). When we compared the usual and optimal treatment groups, a significant difference was observed in the symptoms and signs of fever and tachycardia (*P*-values of 0.02 and 0.03, respectively) (Table II). Furthermore, a significant difference was observed in the diagnosis of aspiration or respiratory tract infections, including pneumonia (*P* < 0.01) (Table II). These findings indicated that aspiration or respiratory tract infections significantly contributed to the decreased antibiotic use.

**Table II:** Frequency of antibiotic treatment in the usual and optimal treatment groups

	Total	Usual treatment group		Optimal treatment group		*P*-value
		*N*	Antibiotic treatment (%)	*N*	Antibiotic treatment (%)	
Chief symptoms and signs						
Fever	47	19	11 (57.9)	28	6 (21.4)	0.02
Nausea or poor appetite	36	20	1 (5)	16	0 (0)	1
Dyspnoea, decrease in percutaneous oxygen saturation, or increased sputum production	28	13	7 (53.8)	15	9 (60)	1
Fatigue	28	4	0 (0)	24	4 (16.7)	1
Vomiting	21	8	3 (37.5)	13	4 (30.8)	1
Abdominal fullness	17	10	6 (60)	7	1 (14.3)	0.13
Convulsion	7	6	0 (0)	1	0 (0)	ND
Tachycardia	7	4	4 (100)	3	0 (0)	0.03
Others	34	15	5 (33.3)	19	6 (31.6)	1
Total	225	99	37 (37.4)	126	30 (23.8)	0.03

Diagnoses						
Aspiration or respiratory tract infection, including pneumonia	76	27	18 (66.7)	49	16 (32.7)	<0.01
Paralytic ileus or gastric distension	56	27	7 (25.9)	29	4 (13.8)	0.32
Dehydration	25	6	0 (0)	19	0 (0)	ND
Seizure	7	5	0 (0)	2	0 (0)	ND
Gastroenteritis	6	2	0 (0)	4	0 (0)	ND
Sepsis and shock	5	3	3 (100)	2	2 (100)	ND
Cardiac failure	3	2	0 (0)	1	0 (0)	ND
Urinary tract infection	2	1	1 (100)	1	1 (100)	ND
Others	26	13	3 (23.1)	13	5 (38.5)	0.67
Total	206	86	32 (37.2)	120	28 (23.3)	0.04

*N*, number of patients; ND, not detected.

Other chief symptoms and signs included chill, facial pallor, poor appetite, sore throat, thirst, stridor, chest pain, nausea, diarrhoea, loose stool, abdominal pain, focal pain, hypouresis, tracheorrhagia, haematemesis, metrorrhagia, decreased level of consciousness, impaired hearing, abnormal cardiac rhythm, increase of gastric residual volumes, reduction of blood pressure, abnormal laboratory value, and medication misuse.

Other diagnoses included mycoplasma infection, peritonitis, dermatitis, arthritis, renal failure, otitis media, empyema, cellulitis, psychogenic reaction, constipation, anaemia, gastric mucosal damage, viral infection, respiratory failure, drug-induced hypotension, genital bleeding, tracheal bleeding, thermoregulation disorder, and bedsores.

When we evaluated the consequences of optimal treatment in patients, no significant differences were observed in the maximum fever level during the event, fever duration during the event, and number of intravenous infusion treatment days between the usual and optimal treatment groups (Table III). Conversely, significant differences were observed in peak C-reactive protein (CRP) level during the event between the usual and the optimal treatment groups (Table III). These findings indicated that treatment based on antimicrobial stewardship principles had been performed with certainty and precisely and did not affect patients.

**Table III:** Comparison of patient conditions and laboratory data between the usual and optimal treatment groups

	Usual treatment group	Optimal treatment group	*P*-value
Maximum fever level during the event (degrees C)			
Number of patients	34	49	
Mean	38.4	38.5	0.38
Range	37.1–40.8	37.2–40.7	
Fever duration (day)			
Number of patients	34	49	
Mean	2.85	2.55	0.6
Range	0–12	0–15	
Number of intravenous drip infusion treatment days (day)			
Number of patients	86	120	
Mean	13.9	14.5	0.8
Range	1–60	1–176	

White blood cell count at the initiation of drip infusion treatment (102/μL)			
Number of patients	82	119	
Mean	103.8	115.4	0.16
Range	25–325	39–299	
Proportion of neutrophil cell number at the initiation of drip infusion treatment (%)			
Number of patients	81	119	
Mean	76.3	76.6	0.87
Range	39–96.5	41.1–96.7	
Peak C-reactive protein level during the event (mg/L)			
Number of patients	82	119	
Mean	37.3	62.1	<0.01
Range	0.03–30.7	0.01–31.1	

**Figure 1:**
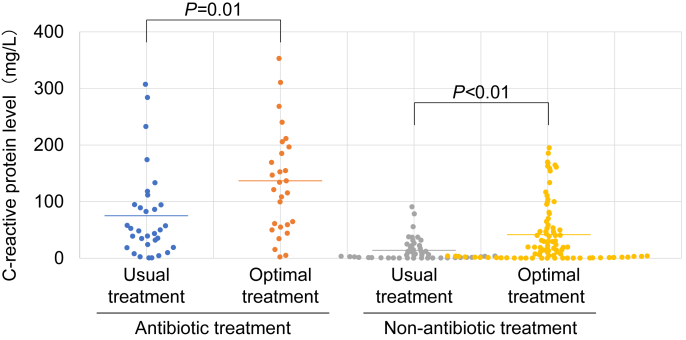
Peak C-reactive protein (CRP) levels during the event observed in this study A comparison of the usual treatment group and the optimal treatment group showed that peak CRP levels (mg/L) during the event were significantly different both with or without antibiotic treatment. The solid lines represent the mean levels for each group.

When we also evaluated the potency of laboratory data due to optimal treatment, significant differences were observed in the peak CRP levels during the events between the usual and optimal treatment groups (*P* < 0.05 for both antibiotic use and non-use) (Table IV and Figure 1). In particular, we found that antibiotic use decreased in events with CRP values < 100 mg/L and antibiotic-free use increased in events with CRP values 100–200 mg/L (Figure 1). Next, to more closely analyse the diagnostic significance of peak CRP levels, we evaluated bacterial culture results. However, no significant trends were observed between peak CRP levels and bacterial culture or antibiotic susceptibility test results (Supplemental Tables I, II and III). These findings indicated that CRP levels support but do not replace clinical judgement.

**Table IV:** Comparison of laboratory data between the usual and optimal treatment groups in the antibiotic use or non-use

	Antibiotic treatment		*P*-value	Non-antibiotic treatment		*P*-value
	Usual treatment group	Optimal treatment group		Usual treatment group	Optimal treatment group	
White blood cell count at the initiation of drip infusion treatment (102/μL)						
Mean	128.1	143.1	0.41	88.3	106.9	0.01
Range	41–325	39–299		25–214	46–269	
Proportion of neutrophil cell number at the initiation of drip infusion treatment (%)						
Mean	79.1	78.6	0.87	74.5	76	0.47
Range	55.1–96.5	52.1–94.1		39–94.8	41.1–95.3	
Peak C-reactive protein level during the event (mg/L)						
Mean	74.5	132	0.01	13.5	40.6	<0.01
Range	0.06–30.7	0.22–31.1		0.03–9.08	0.01–19.5

Taken together, these findings suggested that incidents with CRP values 100–200 mg/L may not necessarily be associated with antibiotic treatment in these long-term care wards and that early determination of the focus of infection may lead to appropriate antibiotic use, especially in the diagnosis of aspiration or respiratory tract infections.

## Discussion

### Clinician-level variability

Antimicrobial stewardship can be framed by the ‘six Ds’: diagnosis, drug, dose, duration, de-escalation, and debridement/drainage [6]. We believe this study may represent an important first step in antimicrobial stewardship in long-term care wards, helping to determine whether or not to prescribe antibiotics after diagnosis. However, clinician-level variability in antimicrobial prescriptions is known [7]. Pandolfo *et al.* reported that clinicians’ perceptions of the need for antibiotics were strongly influenced by beliefs that antibiotics would protect patients from deterioration and themselves from the ethical and legal consequences of undertreatment [8]. They also suggested that efforts to improve antibiotic stewardship should consider clinicians’ desire to defend themselves with a prescription, and rapid molecular microbiology with appropriate communication may diminish clinicians’ fear of not prescribing or using narrower-spectrum antibiotics [8]. We approve on these notions, but we believe that working with medical staff, as in this study, may help address this variability among clinicians.

### Contribution of the intrapulmonary devices

We believe that the decreased antibiotic use in aspiration or respiratory tract infections may be due to the contribution of the intrapulmonary devices that enhance airway clearance by the drainage of sputum; mechanical insufflation–exsufflation, intrapulmonary percussive ventilation, and high-frequency chest wall compression. These devices, which have been used during the study period, have been used in individuals with neuromuscular weakness to augment coughing and/or help clear secretions, as well as in patients who are weak in an acute-care setting due to difficulty in coughing and clearing mucous from the airways, resulting in a decreased risk of choking, recurring chest infections, and ongoing lung disease [9]. Although there is currently no evidence-based report for the use of these devices [10], we postulate that employing these devices to drain sputum may result in decreased antimicrobial use in aspiration or respiratory tract infections in individuals with advanced neuromuscular diseases and severe motor and intellectual disabilities.

### Limitations of the study

Our study had some limitations. Only two clinicians made the decisions to prescribe and to initiate or continue antibiotics, resulting in low variability, although clinician-level variability is often observed in antimicrobial prescriptions. Furthermore, we were unable to evaluate urinary tract infections, which occur frequently in patients with advanced neuromuscular diseases or severe motor or intellectual disabilities because there were only two cases of urinary tract infection among the 206 intravenous infusions studied. Moreover, our study could not be grouped, such as double-blind or randomized controlled trials, owing to a single-centre study design with its small sample size. Our anteroposterior grouping was thought to have a cognitive bias. Additionally, this study lacked microbiological data and long-term clinical outcome measures. Therefore, the appropriate prescription and optimal use of antimicrobials in long-term care wards require extensive research, including the involvement of various clinicians, the accumulation of treated cases and microbiological data, a long follow-up period, and large-scale, multicentre, grouped study designs.

In conclusion, we described the factors to be considered for the appropriate prescription and optimal use of antimicrobials in long-term care wards. Judging the requirement for antibiotics by combining patient conditions with CRP levels may assist in reducing the disproportionate use of antibiotics.

## CRediT authorship contribution statement

**M. Funato:** Writing – review & editing, Writing – original draft, Project administration, Methodology, Investigation, Formal analysis, Data curation, Conceptualization. **R. Iwata:** Writing — review & editing, Project administration, Investigation, Data curation. **K. Yasuda:** Writing – review & editing, Supervision, Project administration, Investigation, Data curation.

## Ethics statement

This study was approved by the Ethics Committee of Nagara Medical Center (approval number: ID 2023–11). This study was evaluated using an opt-out consent approach, which means that patients are included in the retrospective study unless they or the parents of the patients with intellectual disabilities express a desire to be excluded, based on the patient’s low risk and the potential benefit from antimicrobial stewardship.

## Funding sources

This study was supported in part by 10.13039/501100001691JSPS KAKENHI Grant Number JP22K07836.

## Conflicts of interest statement

The authors have no conflicts of interest to declare.

## References

[1] Gu G.Y., Chen M., Pan J.C., Xiong X.L. (2023). Risk of multi-drug-resistant organism acquisition from prior bed occupants in the intensive care unit: a meta-analysis. J Hosp Infect.

[2] Vicentini C., Libero G., Cugudda E., Gardois P., Zotti C.M., Bert F. (2024). Barriers to the implementation of antimicrobial stewardship programmes in long-term care facilities: a scoping review. J Antimicrob Chemother.

[3] Bocquier A., Erkilic B., Babinet M., Pulcini C., Agrinier N. (2024). ORANEAT Study Group. Resident-, prescriber-, and facility-level factors associated with antibiotic use in long-term care facilities: a systematic review of quantitative studies. Antimicrob Resist Infect Control.

[4] Japanese Ministry of Health, Labour and Welfare (2017). https://www.mhlw.go.jp/file/06-Seisakujouhou-10900000-Kenkoukyoku/0000166612.pdf.

[5] Kanda Y. (2013). Investigation of the freely available easy-to-use software 'EZR' for medical statistics. Bone Marrow Transplant.

[6] Morency-Potvin P., Schwartz D.N., Weinstein R.A. (2016). Antimicrobial Stewardship: How the Microbiology Laboratory Can Right the Ship. Clin Microbiol Rev.

[7] Daneman N., Gruneir A., Bronskill S.E., Newman A., Fischer H.D., Rochon P.A. (2013). Prolonged antibiotic treatment in long-term care: role of the prescriber. JAMA Intern Med.

[8] Pandolfo A.M., Horne R., Jani Y., Reader T.W., Bidad N., Brealey D. (2022). INHALE WP2 Study Group. Understanding decisions about antibiotic prescribing in ICU: an application of the Necessity Concerns Framework. BMJ Qual Saf.

[9] Chatwin M., Wakeman R.H. (2023). Mechanical Insufflation-Exsufflation: Considerations for Improving Clinical Practice. J Clin Med.

[10] Morrow B., Argent A., Zampoli M., Human A., Corten L., Toussaint M. (2021). Cough augmentation techniques for people with chronic neuromuscular disorders. Cochrane Database Syst Rev.

