# Supplementary material for: Consideration for the appropriate use of antimicrobials in long-term care wards
Source: Infect Prev Pract. 2026 Apr 12;8(2):100539. doi: 10.1016/j.infpip.2026.100539 (PMC13187596; doi:10.1016/j.infpip.2026.100539)
Supplement: Supplementary file 1 — Multimedia component 1 [file mmc1.pdf]

Supplemental Table I Bacterial culture results in this study

Peak C-reactive protein level during the event (mg/L)	Diagnoses	Antibiotic treatment				Non-antibiotic treatment			
		Usual treatment group		Optimal treatment group		Usual treatment group		Optimal treatment group	
		Number of patients	Bacterial culture	Number of patients	Bacterial culture	Number of patients	Bacterial culture	Number of patients	Bacterial culture
>200	Aspiration and respiratory tract infection including pneumonia	2	Escherichia coli ESBL (1), Klebsiella pneumoniae (2), MRSA (1), Proteus mirabilis (1), Group C Streptococcus (1)	5	Acinetobacter baumann/haemolyticus (2), Elizabethkingia meningoseptica (1), Escherichia coli ESBL (2), Haemophilus influenzae (2), Moraxella catarrhalis (1), MRSA (1), Nonfermenter species (1), Pseudomonas aeruginosa (2), Serratia marcescens (1), Staphylococcus aureus (2), Streptococcus pneumoniae (1)	0		0	
	Paralytic ileus and gastric distension	0		0		0		0	
	Dehydration	0		0		0		0	
	Seizure	0		0		0		0	
	Gastroenteritis	0		0		0		0	
	Sepsis and shock	0		1	Serratia marcescens (1)	0		0	
	Cardiac failure	0		0		0		0	
	Urinary tract infection	0		0		0		0	
	Others	1	Enterococcus spiceis (1), Candida glabrata (1)	0		0		0	
100-200	Aspiration and respiratory tract infection including pneumonia	3	Acinetobacter baumann/haemolyticus (1), Escherichia coli ESBL (2), Klebsiella pneumoniae (2), Providencia stuartii (1), Pseudomonas aeruginosa (1)	7	Acinetobacter baumann/haemolyticus (1), Alcaligenes faecalis (1), Citrobacter koseri (1), Escherichia coli ESBL (1), Haemophilus influenzae (1), Morganella morganii (1), Pseudomonas aeruginosa (2), Stenotrophomonas maltophilia (1), Group C Streptococcus (1), Streptococcus agalactiae (1), Streptococcus pneumoniae (2), Candida albicans (1), Candida glabrata (1)	0		9	Acinetobacter baumann/haemolyticus (2), Haemophilus influenzae BLNAR (1), Moraxella catarrhalis (2), MRSA (2), Proteus mirabilis (1), Pseudomonas aeruginosa (4), Serratia marcescens (2), Staphylococcus aureus (1), Stenotrophomonas maltophilia (1), Group C Streptococcus (1), Streptococcus pneumoniae (4)
	Paralytic ileus and gastric distension	0		1		0		1	
	Dehydration	0		0		0		0	
	Seizure	0		0		0		0	
	Gastroenteritis	0		0		0		0	
	Sepsis and shock	1		1		0		0	
	Cardiac failure	0		0		0		0	
	Urinary tract infection	0		1	Klebsiella pneumoniae (1)	0		0	
	Others	0		1		0		3	
<100	Aspiration and respiratory tract infection including pneumonia	13	Acinetobacter baumann/haemolyticus (2), Elizabethkingia meningoseptica (1), Klebsiella pneumoniae (2), Moraxella catarrhalis (1), MRSA (1), Proteus mirabilis (1), Pseudomonas aeruginosa (5), Staphylococcus aureus (1), Streptococcus agalactiae (2), Streptococcus pneumoniae (3), Candida albicans (1), Candida glabrata (2), Candida krusei (1)	4	Acinetobacter baumann/haemolyticus (1), Burkholderia cepacia (1), Moraxella catarrhalis (2), MRSA (1), Nonfermenter species (1), Pseudomonas aeruginosa (1), Staphylococcus aureus (2), Streptococcus pneumoniae (1)	9	Morganella morganii (1), MRSA (1), Pseudomonas aeruginosa (1), Candida glabrata (1)	24	Acinetobacter baumann/haemolyticus (1), Citrobacter koseri (1), Elizabethkingia meningoseptica (3), Enterobacter cloacae (1), Escherichia coli ESBL (2), Haemophilus influenzae (1), Klebsiella ozaenae (1), Klebsiella pneumoniae (2), Moraxella catarrhalis (3), Morganella morganii (1), MRSA (2), Proteus mirabilis (2), Providencia stuartii (1), Pseudomonas aeruginosa (12), Serratia marcescens (5), Staphylococcus aureus (4), Stenotrophomonas maltophilia (1), Group C Streptococcus (3), Group G Streptococcus (4), Streptococcus agalactiae (3), Streptococcus pneumoniae (3), Candida albicans (3), Candida parapsilosis (1)
	Paralytic ileus and gastric distension	7		3		18 (2)		24	
	Dehydration	0		0		6		19	
	Seizure	0		0		5		1 (1)	
	Gastroenteritis	0		0		2		4	
	Sepsis and shock	2	Streptococcus pneumoniae (1)	0		0		0	
	Cardiac failure	0		0		2		1	
	Urinary tract infection	1	Escherichia coli (1)	0		0		0	
	Others	2	C-Streptococcus (1)	4	Staphylococcus capitis (1)	8 (2)		5	

MRSA; methicillin-resistant Staphylococcus aureus, ESBL; Extended Spectrum β-Lactamase; BLNAR; β-lactamase non-producing ampicillin-resistant

Note: Numbers in parentheses indicate the number of untested patients.
