# Supplementary material for: Consideration for the appropriate use of antimicrobials in long-term care wards
Source: Infect Prev Pract. 2026 Apr 12;8(2):100539. doi: 10.1016/j.infpip.2026.100539 (PMC13187596; doi:10.1016/j.infpip.2026.100539)
Supplement: Supplementary file 3 — Multimedia component 3 [file mmc3.pdf]

Supplemental Table III Antibiotic susceptibility test results in aspiration and respiratory tract infections without antibiotic treatment

	Usual treatment group								
Peak C-reactive protein level during the event (mg/L)	<100								
Bacterial species	Morganella morganii			MRSA			Pseudomonas aeruginosa		
Antimicrobials	S	I	R	S	I	R	S	I	R
MPIPC						1			
CVA/AMPC									
AZT	1						5		
IPM/CS						1	1		
CP									
CL							1		
RFP				1					
FOM			1			1			1
EM						1			
LVFX	1					1	1		
CAM									
CCL			1						
GM	1					1	1		
AZM									
CPFX							1		
SBT/CPZ	1						1		
CEZ			1			1			
CTX									
CMZ	1								
CFDN						1			
TEIC				1					
CLDM						1			
TOB							1		
PCG						1			
ST	1			1					
ABK				1					
VCM				1					
CTM			1			1			
ABPC			1			1			
AMK	1						1		
CZOP							1		
FMOX	1					1			
CFPN-PI	1								
PIPC	1						1		
CFPM	1						1		
MINO	1					1			
CDTR-PI									
MEPM	1						1		
CAZ		1					1		
SBT/ABPC			1			1			
CTRX	1								
DRPM							1		
LZD				1					
TAZ/PIPC	1						1		

	Optimal treatment group																	
Peak C-reactive protein level during the event (mg/L)	<100																	
Bacterial species	Acinetobacter baumann/haemolyticus			Citrobacter koseri			Elizabethkingia meningoseptica			Enterobacter cloacae			Escherichia coli ESBL			Haemophilus influenzae		
Antimicrobials	S	I	R	S	I	R	S	I	R	S	I	R	S	I	R	S	I	R
MPIPC																		
CVA/AMPC																1		
AZT				1					3	1					2			
IPM/CS	1			1					3	1			2					
CP																1		
CL	1																	
RFP																1		
FOM																		
EM																		
LVFX	1			1			3			1					2			
CAM																		1
CCL				1								1			2		1	
GM	1			1					3	1			2					
AZM																		
CPFX	1						2	1										
SBT/CPZ	1			1						1					2			
CEZ				1								1			2			
CTX				1						1					2	1		
CMZ				1								1	2					
CFDN																		
TEIC																		
CLDM																		
TOB	1								3									
PCG																		
ST	1			1			3			1			2			1		
ABK																		
VCM																		
CTM				1								1			2		1	
ABPC						1					1				2	1		
AMK	1			1					3	1			2					
CZOP	1								3									
FMOX				1								1	2					
CFPN-PI				1						1					2			
PIPC	1			1					3	1					2			
CFPM	1			1					3	1					2			
MINO	1			1			3			1			2			1		
CDTR-PI																1		
MEPM	1			1					3	1			2			1		
CAZ	1			1					3	1					2			
SBT/ABPC				1						1					2	1		
CTRX				1								1			2	1		
DRPM	1																	
LZD																		
TAZ/PIPC				1			1	1	1	1			2					

	Optimal treatment group																	
Peak C-reactive protein level during the event (mg/L)	<100																	
Bacterial species	Klebsiella ozaenae			Klebsiella pneumoniae			Moraxella catarrhalis			Morganella morganii			MRSA			Proteus mirabilis		
Antimicrobials	S	I	R	S	I	R	S	I	R	S	I	R	S	I	R	S	I	R
MPIPC															2			
CVA/AMPC																		
AZT	1			2						1						2		
IPM/CS	1			2											2			
CP							3											
CL																		
RFP													2					
FOM													1		1			
EM										1					2			
LVFX	1			2			3						1		1	2		
CAM							3											
CCL	1			2			3					1				2		
GM	1			2						1			1		1	2		
AZM																		
CPFX							3											
SBT/CPZ	1			2						1						2		
CEZ	1			2								1			2	2		
CTX	1			2			3									2		
CMZ	1			2						1						2		
CFDN															2			
TEIC													2					
CLDM															2			
TOB																		
PCG															2			
ST	1			2						1			2			2		
ABK													2					
VCM													2					
CTM	1			2								1			2	2		
ABPC	1											1			2	2		
AMK	1			2						1						2		
CZOP																		
FMOX	1			2						1					2	2		
CFPN-PI				2														
PIPC		1			1	1				1						2		
CFPM	1			2						1						2		
MINO	1			2						1			2				2	
CDTR-PI																		
MEPM	1			2						1						2		
CAZ	1			2							1					2		
SBT/ABPC	1			1	1							1			2	2		
CTRX	1			2			3			1						2		
DRPM																		
LZD													2					
TAZ/PIPC	1			2						1						2		

	Optimal treatment group																	
Peak C-reactive protein level during the event (mg/L)	<100																	
Bacterial species	Providencia stuartii			Pseudomonas aeruginosa			Serratia marcescens			Staphylococcus aureus			Stenotrophomonas maltophilia			Group C Streptococcus		
Antimicrobials	S	I	R	S	I	R	S	I	R	S	I	R	S	I	R	S	I	R
MPIPC										4								
CVA/AMPC																		
AZT	1			12			5											
IPM/CS				10		2				4								
CP																3		
CL				12														
RFP										4								
FOM				1	1	10				4								
EM										2		2				1		2
LVFX			1	10		2	4		1	3		1	1			3		
CAM																		
CCL			1						5									
GM			1	11	1		5			2		2						
AZM																1		2
CPFX				10		2												
SBT/CPZ	1			10	2		5											
CEZ			1						5	4								
CTX			1													3		
CMZ	1						5											
CFDN										4								
TEIC										4								
CLDM										2		2				3		
TOB				12														
PCG										4						3		
ST	1						5			4			1					
ABK										4								
VCM										4						3		
CTM			1						5	4								
ABPC			1					1	4	4						3		
AMK	1			12			5											
CZOP				12											1	3		
FMOX		1					4		1	4								
CFPN-PI							3											
PIPC	1			10	2		5											
CFPM	1			12			5									3		
MINO			1				4	1		4			1			1		2
CDTR-PI																		
MEPM	1			10		2	5									3		
CAZ	1			12			5								1			
SBT/ABPC		1						1	4	4								
CTRX	1						5									3		
DRPM				11	1													
LZD										4								
TAZ/PIPC	1			10	2		4	1										

	Optimal treatment group								
Peak C-reactive protein level during the event (mg/L)	<100								
Bacterial species	Group G Streptococcus			Streptococcus agalactiae			Streptococcus pneumoniae		
Antimicrobials	S	I	R	S	I	R	S	I	R
MPIPC									
CVA/AMPC							3		
AZT									
IPM/CS									
CP	4			3			3		
CL									
RFP							3		
FOM									
EM	4			3					3
LVFX	4			3			3		
CAM									
CCL									
GM									
AZM	4			3					3
CPFX									
SBT/CPZ									
CEZ									
CTX	4			3			3		
CMZ									
CFDN									
TEIC									
CLDM	4			3					3
TOB									
PCG	4			3			3		
ST							3		
ABK									
VCM	4			3			3		
CTM									3
ABPC	4			3					
AMK									
CZOP	4			3			3		
FMOX									
CFPN-PI									
PIPC									
CFPM	4			3			3		
MINO			4	3		1	1	1	1
CDTR-PI							2	1	
MEPM	4			3				3	
CAZ									
SBT/ABPC									
CTRX	4			3			3		
DRPM									
LZD									
TAZ/PIPC									

	Optimal treatment group																	
Peak C-reactive protein level during the event (mg/L)	100-200																	
Bacterial species	Acinetobacter baumann/haemolyticus			Haemophilus influenzae BLNAR			Moraxella catarrhalis			MRSA			Proteus mirabilis			Pseudomonas aeruginosa		
Antimicrobials	S	I	R	S	I	R	S	I	R	S	I	R	S	I	R	S	I	R
MPIPC												2						
CVA/AMPC						1												
AZT													1			3		1
IPM/CS	2											2				4		
CP	2			1			2											
CL	2															4		
RFP				1						2								
FOM										1		1						4
EM												2						
LVFX	2			1			2					2	1			3	1	
CAM						1	2											
CCL						1	2						1					
GM	2									2			1			3	1	
AZM																		
CPFX	2						2									2	1	1
SBT/CPZ	2												1			3		1
CEZ												2	1					
CTX				1			2						1					
CMZ													1					
CFDN												2						
TEIC										2								
CLDM												2						
TOB	2															4		
PCG												2						
ST	1		1	1						2			1					
ABK										2								
VCM										2								
CTM						1						2	1					
ABPC					1							2	1					
AMK	2												1			4		
CZOP	2															3		1
FMOX												2	1					
CFPN-PI													1					
PIPC	2												1			3		1
CFPM	2												1			3	1	
MINO	2			1						1	1				1			
CDTR-PI				1														
MEPM	2			1									1			4		
CAZ	2												1			3		1
SBT/ABPC						1						2	1					
CTRX				1			2						1					
DRPM	2															4		
LZD										2								
TAZ/PIPC													1			3		1

	Optimal treatment group														
Peak C-reactive protein level during the event (mg/L)	100-200														
Bacterial species	Serratia marcescens			Staphylococcus aureus			Stenotrophomonas maltophilia			Group C Streptococcus			Streptococcus pneumoniae		
Antimicrobials	S	I	R	S	I	R	S	I	R	S	I	R	S	I	R
MPIPC				1											
CVA/AMPC													4		
AZT	2														
IPM/CS				1											
CP										1			4		
CL															
RFP				1									4		
FOM				1											
EM						1						1			4
LVFX	2			1			1			1			4		
CAM															
CCL			2												
GM	2					1									
AZM												1			4
CPFX															
SBT/CPZ		2													
CEZ			2	1											
CTX										1			4		
CMZ	2														
CFDN				1											
TEIC				1											
CLDM						1				1					4
TOB															
PCG				1						1			4		
ST	2			1			1						1	3	
ABK				1											
VCM				1						1			4		
CTM			2	1											4
ABPC			2	1						1					
AMK	1	1													
CZOP									1	1			4		
FMOX		2		1											
CFPN-PI		1													
PIPC	1	1													
CFPM	2									1			4		
MINO	2			1			1					1		1	3
CDTR-PI													3	1	
MEPM	2									1			1	3	
CAZ	2							1							
SBT/ABPC			2	1											
CTRX	1		1							1			4		
DRPM															
LZD				1											
TAZ/PIPC	1	1													

S; susceptible, I; intermediate, R; resistant, MRSA; methicillin-resistant Staphylococcus aureus, ESBL; Extended Spectrum β-Lactamase, BLNAR; β-lactamase non-producing ampicillin-resistant, MPIPC; oxacillin, CVA/AMPC; clavulanic acid/amoxicillin, AZT; aztreonam, IPM/CS; imipenem/cilastatin, CP; chloramphenicol, CL; colistin, RFP; rifampicin, FOM; fosfomycin, EM; erythromycin, LVFX; levofloxacin, CAM; clarithromycin, CCL; cefaclor, GM; gentamicin, AZM; azithromycin, CPFX; ciprofloxacin, SBT/CPZ; sulbactam/cefoperazone, CEZ; cefazolin, CTX, cefotaxime, CMZ; cefmetazole, CFDN; cefdinir, TEIC; teicoplanin, CLDM; clindamycin, TOB; tobramycin, PCG; benzylpenicillin（penicillin G）, ST; sulfamethoxazole-trimethoprim, ABK; arbekacin, VCM; vancomycin, CTM; cefotiam, ABPC; ampicillin, AMK; amikacin, CZOP; cefozopran, FMOX; flomoxef, CFPN-PI; cefcapene pivoxil, PIPC; piperacillin, CFPM; cefepime, MINO; minocycline, CDTR-PI; cefditoren pivoxil, MEPM; meropenem, CAZ; ceftazidime, SBT/ABPC; sulbactam/ampicillin, CTRX; ceftriaxone, DRPM; doripenem, LZD; linezolid; TAZ/PIPC; tazobactam/piperacillin
